# Toll-like receptor 4 (TLR4) expression is correlated with T2* iron deposition in response to doxorubicin treatment: cardiotoxicity risk assessment

**DOI:** 10.1038/s41598-020-73946-9

**Published:** 2020-10-12

**Authors:** Nelu-Mihai Trofenciuc, Aurora Diana Bordejevic, Mirela Cleopatra Tomescu, Lucian Petrescu, Simina Crisan, Oliviana Geavlete, Alexandru Mischie, Alexandru Fica Mircea Onel, Alciona Sasu, Adina Ligia Pop-Moldovan

**Affiliations:** 1 “Victor Babeş” University of Medicine and Pharmacy, Timisoara, Romania; 2 Institute of Cardiovascular Disease, Timisoara, Romania; 3 Timisoara Municipal Emergency Clinical Hospital, Timisoara, Romania; 4 “Carol Davila” University of Medicine and Pharmacy, Bucharest, Romania; 5 Institute of Cardiovascular Diseases ’Prof. Dr. C. C. Iliescu, Bucharest, Romania; 6 Centre Hospitalier de Montluçon, Invasive Cardiology Unit, Cardiology Department, Montluçon, France; 7 “Vasile Goldis” Western University of Arad, Arad, Romania; 8 Arad County Clinical Emergency Hospital, Hematology Department, Arad, Romania; 9 Arad County Clinical Emergency Hospital, Cardiology Department, Arad, Romania

**Keywords:** Genetics, Cardiology, Medical research, Molecular medicine, Oncology, Cancer, Cancer therapy, Chemotherapy

## Abstract

Although doxorubicin (Dox) is an effective antitumor antibiotic in the anthracycline class, it often induces the undesirable side effect of cardiomyopathy leading to congestive heart failure, which limits its clinical use. The primary goal of this study is to evaluate a reliable translational method for Dox-induced cardiotoxicity (CTX) screening, aiming to identify a high-risk population and to discover new strategies to predict and investigate this phenomenon. Early identification of the presence of iron deposits and genetic and environmental triggers that predispose individuals to increased risk of Dox-induced CTX (e.g., overexpression of Toll-like receptor 4 (TLR4)) will enable the early implementation of countermeasure therapy, which will improve the patient’s chance of survival. Our cohort consisted of 25 consecutive patients with pathologically confirmed cancer undergoing Dox chemotherapy and 12 control patients. The following parameters were measured: serum TLR4 (baseline), serum transferrin (baseline and 6-week follow-up) and iron deposition (baseline and 6-week follow-up). The average number of gene expression units was 0.121 for TLR4 (range 0.051–0.801). We subsequently correlated serum TLR4 levels in our cohort with myocardial iron overload using the cardiac magnetic resonance (CMR) T2* technique, the ventricular function (% ejection fraction, %EF) and serum transferrin levels. There is a strong negative linear relationship between serum TLR4 and CMR T2* values (r =  − 0.9106, ****P < 0.0001). There is also a linear correlation (either positive or negative) with EF and transferrin; no established relationship related to the sex of the patients was found. Patients with elevated serum TLR4 at baseline also exhibited an increase in serum transferrin levels and Dox-induced left ventricular dysfunction with a decreased EF (< 50%); this phenomenon was observed in 7 of 25 patients (28%) at the 6-week follow-up. There were no significant differences or correlations based on sex. We concluded that there is a direct relationship between Dox-induced CTX (indicated by elevated serum TLR4) and the times (ms) for T2* (decreases in which correspond to immediate and rapid iron overload).

## Introduction

Doxorubicin (Dox) is an effective antitumor antibiotic in the anthracycline class. However, Dox also induces cardiomyopathy leading to congestive heart failure, thus often limiting its clinical use^1^.

Cardiotoxicity (CTX), including subclinical, acute and late-onset CTX, is a well-known adverse effect of many antitumor agents. Early identification of patients with risk factors associated with CTX is important to ensure prompt clinical intervention and to minimize toxic effects from the antitumor drug.

To maximize the benefits of anthracyclines, a high-risk population needs to be identified, and new strategies to predict and investigate this phenomenon need to be developed for monitoring patients and minimizing toxic side effects^2^.

The etiology of chemotherapy-induced cardiotoxicity (CIC) is multifactorial. Actual traditional methods used in our daily clinical practice to assess for CIC typically include serial measurements of cardiac function and parameters via multimodality imaging techniques, of which the most commonly used is 2D echocardiography to assess the left ventricle ejection fraction (LVEF)^3,4^.

### TLR4

Several Toll-like receptors (TLRs) are expressed in cardiomyocytes, including TLR2 and 4. Through these TLRs, cardiomyocytes can respond to endogenous or exogenous signals that influence pathophysiological responses to dilated cardiomyopathy. Human TLR4 was among the first mammalian Toll-related proteins to be described and is secreted from the endoplasmic reticulum. The expression or activation of TLR4 is upregulated in experimental models of cardiomyopathy and in patients with hypertension and/or clinical heart failure^5^.

TLRs are members of the interleukin-1 receptor family (IL1) and are involved in the ability to react to molecular triggers associated with pathogenic microorganisms. Recent studies have shown that TLRs are activated by endogenous signals, such as heat shock proteins and oxidative stress, which can contribute to congestive heart failure^6^.

During the inflammatory response, TLRs recognize pathogens associated with molecular patterns such as lipopolysaccharides, peptidoglycan, bacterial lipoproteins and oligonucleotides.

Intracellular signaling pathways of TLRs are similar to IL1-like pathways and lead to the nuclear localization of the nuclear factor (NF) kb/Rel-type^7^. Moreover, TLRs are expressed in different organs, such as the lung, brain, kidney, and heart^2,8^.

Oxidative stress is one of the major effects in Dox-induced cardiac dysfunction. In a previous study, we hypothesized and showed that TLRs contribute to the pathogenesis of Dox-induced cardiac dysfunction via an inflammation-induced mechanism^6^. These studies also indicate that free radicals play an important role in Dox-induced CTX.

We must also keep in mind that the TLR family was recently demonstrated to play an important role in ventricular remodeling after myocardial infarction as well as in cardiac specificity^9^, which might drive future research in this field.

### Assessment of iron status in the human body

Adult men and women have approximately 3.5 g and 2.3 g of total body iron, respectively. This iron is dispersed in various organ systems: approximately 65% and 75% are bound to circulating hemoglobin, 22% and 12% are deposited in liver (ferritin and hemosiderin), and 2.9% and 4.6% are bound to various enzymes. For both sexes, approximately 10% of iron is coupled with myoglobin, which is an oxygen-binding protein located in muscle tissue, and only a small percentage of red blood cells bound to transferrin was observed in men (0.1%) and in women (1.3%)^10,11^.

During the processes of infection and inflammation, serum ferritin levels are frequently within the normal range (> 12 μg/L and < 100 μg/L), and iron deficiency can only be excluded by an equivalent measurement of soluble transferrin receptor and at least two acute phase proteins—C-reactive protein and α-1-acid glycoprotein^10,12^.

We define iron overload as increased serum ferritin levels (> 200 μg/L in men and > 150 μg/L in women) and transferrin saturation > 50%.

### Transferrin as an innate immune mediator

The main reason for strict iron regulation is that elemental iron is a highly effective pro-oxidant; as such, its free form stimulates the formation of free radicals, which are very distressing (with harmful potential) to cell membranes, intracellular components and macromolecules.

Iron itself is a key element required for the intracellular accumulation of lipid peroxide molecules and for the initiation of the ferroptosis cascade. Thus, the import, export, storage, and turnover of iron has an important impact on ferroptosis sensitivity. Transferrin and transferrin receptors, which function in iron import from the extracellular environment, are mandatory for the ferroptosis process^13^. Additionally, in support of this connection, suppressing the iron metabolism master regulator IREB2 (*iron-responsive element-binding protein 2*) decreases sensitivity to ferroptosis^14^. To better understand these interrelationships, we specify that the IREB2 gene is involved in encoding the iron-responsive element-binding protein (IRP), which controls the expression of proteins involved in iron metabolism (via binding to iron-responsive elements in the mRNAs of target genes, which includes the transferrin receptor (TFRC; 190010) and the heavy and light ferritin chains (FTH1, 134770 and FTL, 134790, respectively). IREB2, similar to IREB1 (100880), has differing functions in iron-deficient and iron-replete states^15,16^. In a study involving a mouse model, controlled targeted disruption of the Ireb2 gene was followed by dysregulation of iron metabolism in the intestinal mucosa and onset of a de novo neurodegenerative disease (at the central nervous system level)^17^.

### Dox, TLR4, and cardiac iron: the good, the bad and the ugly

Nonapoptotic forms of cell death can trigger a systemic inflammation cascade process through the release of danger-associated molecular patterns (DAMPs), which are recognized by innate immune receptors (IIRs).

Dox-based chemotherapy drugs have an effect on cancer cells by essentially intercalating the target DNA and by disrupting and/or inhibiting topoisomerase II function^18,19^.

Ferroptosis is a recently discovered form of programmed cell death that occurs via lipid peroxidation caused by iron accumulation^20^. Ferroptosis is dependent upon intracellular iron but not upon other metals and is considered morphologically, biochemically and genetically distinct from apoptosis, necrosis and autophagy^14^.

Recent animal studies have provided new data and conclusions that ferroptotic cell death triggers initial inflammatory responses after heart transplantation. It is further postulated that nonapoptotic forms of programmed cell death, such as necroptosis or ferroptosis, result in the release of DAMPs that ultimately result in the initiation of the inflammatory, which is referred to as necroinflammation^21,22^. The precise cell death pathways and signaling events that orchestrate early inflammation after heart transplantation are unknown, as is anthracycline-induced CTX (Dox in particular). Both processes share a similar, if not identical, cardiovascular response (e.g., iron loading of cardiomyocytes, the presence of DAMPs and induced secondary ferroptosis)^22^.

Dox thus plays an important role in this scenario, both directly by activating TLR4-dependent events (a response that is directly proportional to the number of receptors) and indirectly via the inflammatory cascade generated by DAMPs. The common element in all these scenarios is intracellular iron loading.

### CTX assessment

The common clinical parameter indicative of subclinical CTX is usually outlined on cardiac imaging as clinically asymptomatic left ventricular systolic dysfunction (LVSD) with a decrease in the LVEF by > 10% and an overall EF < 50%^23^. LVEF can be calculated based on multigated acquisition (MUGA) radionuclide angiocardiography, echocardiography, or cardiovascular MRI.

However, there is no clear consensus for the definition for early cardiac injury or any other imaging biomarkers beyond those associated with functional assessment. Cardiovascular MRI may identify additional biomarkers that could facilitate a more sensitive and specific identification of CTX^24^.

Recent developments in techniques to assess cardiac function include not only qualitative techniques for tissue level differentiation (subclinical assessment) but also quantitative approaches (clinical assessments, decreased EF).

Several studies have determined that screening for CTX by assessing the EF may be inadequate to detect subclinical disease. Thus, more sensitive methods for the assessment of cardiac structure and function, such as MRI, biomarkers and genetic screening, have a stronger ability to detect the subclinical forms of CTX^25,26^.

## Material and methods

This study was an analytical, multicenter prospective study that took place between 2018 and 2019.

## Subjects

Our cohort included 25 consecutive patients aged 18–65 years old who received Dox treatment for hematological malignancies (leukemia, lymphomas or multiple myeloma) with a survival probability > 6 months and an LVEF > 50% and who provided written informed consent. Exclusion criteria consisted of previous anthracycline therapy, previous radiotherapy, history of heart failure or chronic renal failure, atrial fibrillation or other significant arrhythmias, pregnancy and any past medical history involving iron deficiency or accumulation (including an extended period of iron supplementation).

## Methods

**Figure 1:**
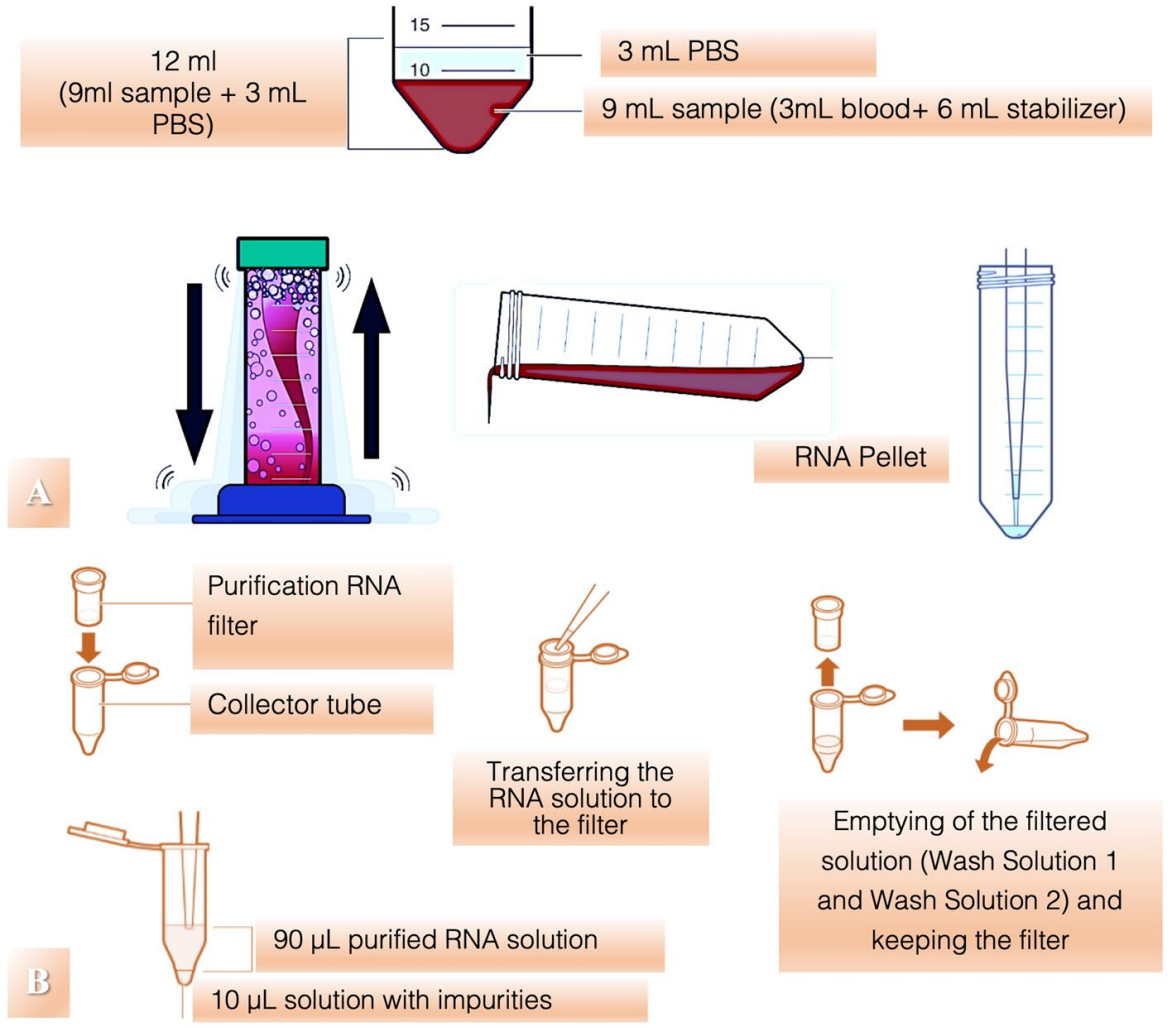
Schematic of the laboratory methodology used. (**A**) RNA isolation procedure. (**B**) RNA purification procedure.

A peripheral blood sample was drawn from all patients who were in the fasting state to assess serum TLR4 gene expression and serum transferrin levels. Gene expression was assessed by qRT-PCR using the following steps: blood collection (3 ml), RNA isolation, cDNA reverse transcription, qRT-PCR and quantification of the relative expression. Patient blood was drawn directly into a Tempus Blood RNA Tube (4342792 Applied Biosystems). Serum transferrin levels were also quantified at follow-up (3 and 6 weeks). A schematic of the whole process is illustrated in (Fig. 1).

**Figure 2:**
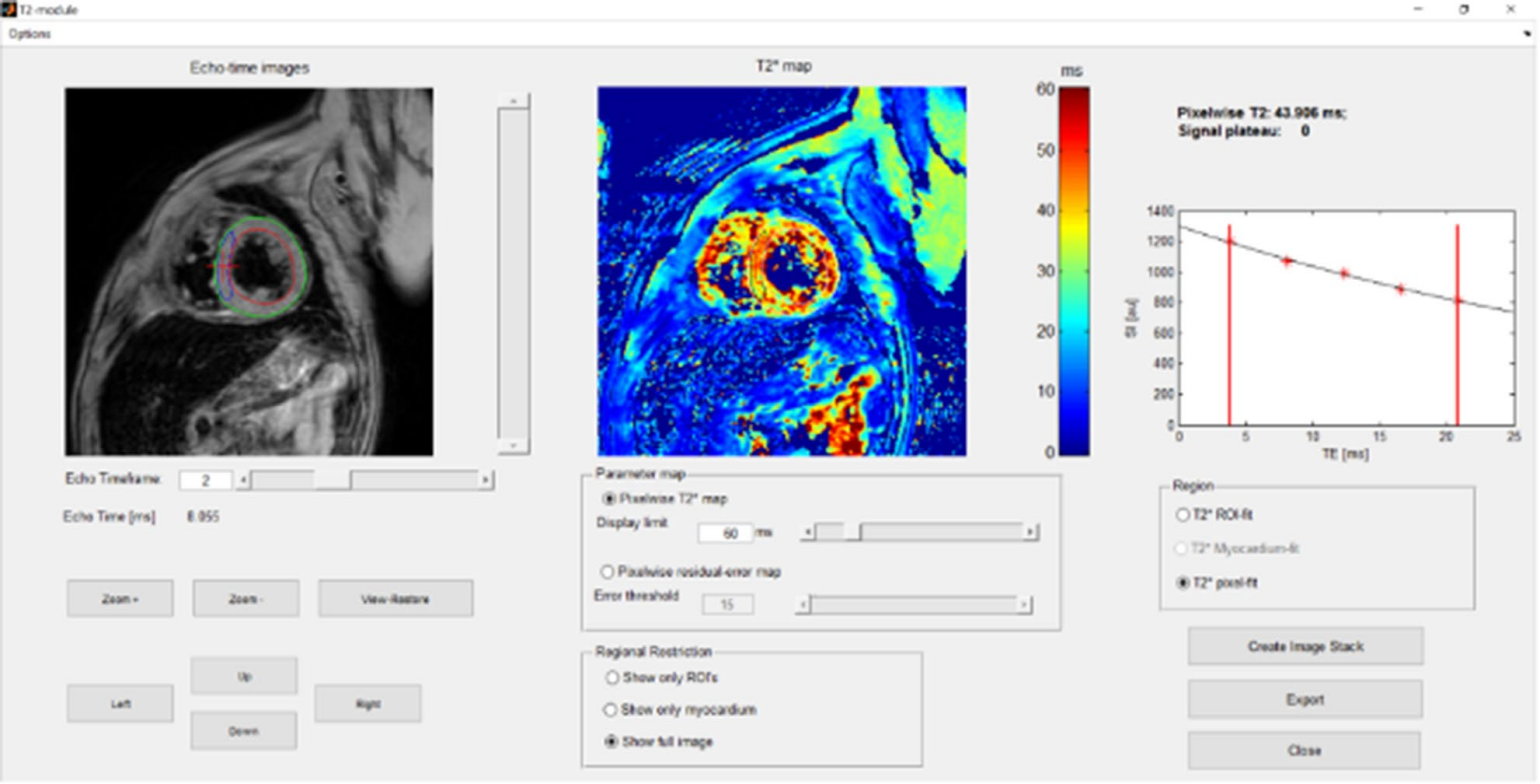
T2* Analysis. + represents the selected region of interest (ROI) for this analyzed case.

At enrollment, all patients were clinically evaluated, and a CMR examination was performed. The CMR parameters determined were those of LV systolic function (EF%) and T2* determination of iron overload in a specific septal region of interest (ROI) (Fig. 2). All image analyses were performed using Segment software version 3.0 R7946 (Academic Research Version).

The medical history and physical examination for every patient were also assessed, with specific focus on the signs and symptoms of heart failure as well as the risk factors for cardiovascular disease. The past medical and drug histories were recorded.

Each patient was informed and briefed in advance regarding this study and its implications, and written informed consent was obtained prior to study enrollment. Additionally, this study was approved by the Ethics Committee for Scientific Research of the County Emergency Clinical Hospital from Arad (available upon request from the corresponding author).

Statistical analysis of potential risk factors and correlations (direct or indirect) with the development of early cardiomyopathy as a function of Dox-induced CTX was conducted. Statistical analyses were performed using GraphPad Prism version 8.0.0 for Mac OS X (GraphPad Software, San Diego California USA). Continuous (numeric) variables were expressed as the means ± standard deviation (SD) or medians [quartiles] if not normally distributed; by contrast, categorical variables were reported as counts and percentages. Descriptive and inferential statistical analysis methods were used. Correlations between variables were calculated using the simple Pearson correlation coefficient. All tests were two-tailed, and a *P* value < 0.05 was considered statistically significant.

Following the application of these tests, the main parameters of interest for our study were discussed, and the conclusions were established based on the values of the parameters in question.

### TLR4 quantification

Tempus Blood RNA tubes were used to collect peripheral human blood samples (4342792, Applied Biosystems). Each tube contained 6 ml of stabilizing reagent that ensured RNA stabilization for 4 to 5 days at room temperature or for a minimum of 7 days at 4 °C. RNA was isolated within three days of collection. In each tube, 3 ml of peripheral venous blood was collected from patients^6^.

After isolation, the RNA quantity and quality were measured using a NanoDrop 1000 Spectrophotometer (Thermo Scientific). Before using RNA samples in subsequent experiments, the integrity of the RNA was assessed by running aliquots of the RNA sample on a denaturing agarose gel spiked with ethidium bromide. Only samples that showed clear 28S and 18S rRNA bands were used in cDNA reactions. For cDNA synthesis, we used High Capacity cDNA Reverse Transcription Kits (4368814, Applied Biosystems) according to the manufacturer’s instructions, and the concentration of the RNA template was 25 ng μl^−1^^7,27^.

For qRT-PCR, we used a LightCycler 480 SYBR Green I Master kit and a Thermocycler-µl Light Cycler 480. The primers used were as follows: TLR4, forward TTGAGCAGGTCTAGGGTGATTGAAC and reverse ATGCGGACACACACACTTTCAAATA and GAPDH, forward GCACCGTCAAGGCTGAGAAC and reverse TGGTGAAGACGCCAGTGGA (Shan J.-Y., et al. 2011).

The primer sequences were verified using Primer-BLAST at the National Center for Biotechnology Information (https://www.ncbi.nlm.nih.gov). The primer concentration for qRT-PCR was 10 ng/µl, and 5 µl from cDNA solution was used for each reaction. All samples were assessed in triplicate. Following qRT-PCR, the samples were verified by agarose gel electrophoresis. The data were analyzed using Light Cycler 480 Software, Basic/Advanced Relative Quantitation program (Roche Diagnostics, Mannheim, Germany)^6^.

Continuous data are expressed as numerical values indicated by the means ± standard deviation. The differences between pairs of samples (in mRNA levels) were calculated using the 2-∆∆Ct method, which were considered significant when the mRNA levels varied by a factor of more than 1.8.

### CMR T2*

The T2* values from the ROIs at the mid-left ventricle by the 1.5 T CMR unit for chemotherapy patients and healthy controls were assessed at baseline and at 6 weeks after treatment initiation. The LVEF was also quantified.

T2* quantification was performed in myocardium assessed by a breath-hold and ECG-triggered, spoiled gradient echo sequence with multiple echoes; T2* mapping was performed with a breath-hold multislice gradient echo sequence. A full-thickness ROI was measured in the LV myocardium in both epicardial and endocardial regions. This ROI was located in the septum distant from the cardiac veins, which reduced the sensitivity for obtaining artifacts^28^.

We used this approach as a predetermined standard that hinged on the hypothesis that changes in CTX are represented by the decrease in the EF < 50% or a decrease by more than 10% of the determined initial value^29^.

### Transferrin

Serum transferrin concentration was determined using an Axsymanalyzer (Abbott, Chicago, IL, USA). The assays were performed according to the manufacturer’s instructions.

### Study ethics

All methods were performed in accordance with the relevant guidelines and regulations enforced from the time of study enrollment to the publication of the data obtained. Subjects provided written informed consent, which was obtained before enrollment in the study.

## Results

A total of 25 consecutive oncological patients were included in this study. Subjects in the study and control groups were well matched for age and sex distribution. Out of the 25 patients, 13 (52%) were males and 12 (48%) were females. The mean age was 57.3 years, the mean dose of Dox administered ranged between 100 and 250 mg/m^2^, and the Dox clearance was 1442 ± 111 ml/min/m^2^.

The main demographic and clinical characteristics are presented in Table 1. The studied parameters and their related values are presented in detail in Table 2.

**Table 1:** Characteristics of the study patients. ns- not statistically significant or not applicable. Data are expressed as the mean followed by " ± " mean standard deviation (SD) or as the number of cases found. *P* was obtained by applying an ANOVA test.

Variables	Study cohort	Control group	*P* value
Age (years)	57.3 ± 12.1	55.2 ± 6.8	ns
**Sex**			
Male	13	6	ns
Female	12	6	ns
**Oncological disorder**			
Non-Hodgkin's lymphoma	5	–	ns
Acute myeloid leukemia	3	–	ns
Acute lymphoblastic leukemia	4	–	ns
Breast cancer (BC)	7	–	ns
Solid tumor other than BC	6	–	ns
**Comorbidities**			
Hyperlipidemia	14	6	ns
Diabetes	8	3	ns
Hypertension	11	8	ns

**Table 2:** Studied parameters and their related values. ***, **** indicate statistically significant *P* values (< 0.05). ns indicates not statistically significant or not applicable. Data are expressed as the means ± mean standard deviation (SD). *P* values were obtained via ANOVA.

	TLR4	T2* initial (ms)	T2* 6 weeks (ms)	EF% baseline	EF% 6 weeks	Transferrin baseline	Transferrin 6 weeks
Study group (n = 25)	0.111 ± 0.052	23.91 ± 1.93	22.04 ± 2.74	64.04 ± 6.11	58.56 ± 8.19	90 ± 25.29	108.26 ± 39.25
Control group (n = 12)	0.087 ± 0.050	24.91 ± 1.32	24.58 ± 1.25	66.25 ± 5.21	65.66 ± 5.46	84.46 ± 34.04	86.91 ± 34.92
*P* value	ns	****P < 0.0001		****P < 0.0001		***P < 0.0003	

**Figure 3:**
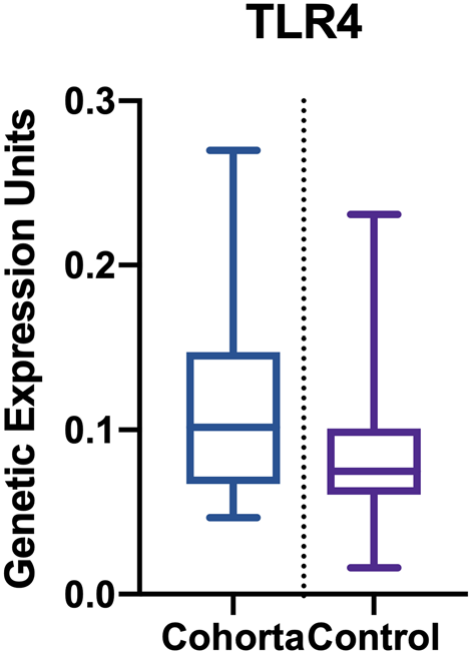
TLR4 determination.

The average number of gene expression units for TLR4 was 0.121 (range 0.051–0.801). The mRNA levels of target genes were normalized to that of GAPDH; the result is presented as relative gene expression. The mean quantity of mRNA extracted was 113.571 ng/µl (Fig. 3).

There is a strong negative linear relationship between TLR4 expression and CMR T2* values (r =  − 0.9106, ****P < 0.0001), where r = maximum simple correlation coefficient (Pearson). There is also a linear correlation (either positive or negative) with EF and transferrin; however, there is no established relationship related to the genetic sex of the patients. The main TLR4 correlations are presented in Table 3.

**Table 3:** TLR4 correlations with study parameters. ***, **** indicate statistically significant *P* values (< 0.05). ns, no statistical significance.

Correlation	TLR4 versus T2* initial	TLR4 versus T2* 6 weeks	TLR4 versus EF% initial	TLR4 versus EF% 6 weeks	TLR4 versus Transferrin initial	TLR4 versus Transferrin 6 Weeks	TLR4 versus Sex
r	− 0.8527	− 0.9106	− 0.6581	− 0.6779	0.6868	0.7628	− 0.2089
CI	− 0.9359 to − 0.6792	− 0.9618 to − 0.7980	− 0.8419 to − 0.3375	− 0.8520 to − 0.3687	0.3830 to 0.8565	0.5114 to 0.8939	− 0.5719 to 0.2225
R^2^	0.7271	0.8293	0.4331	0.4596	0.4717	0.5818	0.04364
*P* value	< 0.0001	< 0.0001	0.0006	0.0004	0.0003	< 0.0001	0.3388
*P* value summary	****	****	***	***	***	****	ns

**Figure 4:**
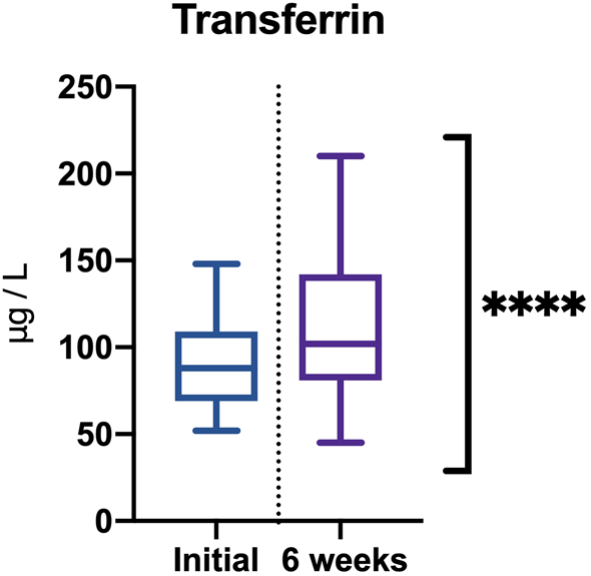
Determination of transferrin levels.

Baseline serum transferrin levels (90 ± 25.87 ηg/ml) were significantly correlated (r = 0.898; *P* < 0.0001) with the 6-week levels(108 ± 40.13 ηg/ ml). There was no sex-based significant difference or correlation for this evaluated parameter (Fig. 4).

**Figure 5:**
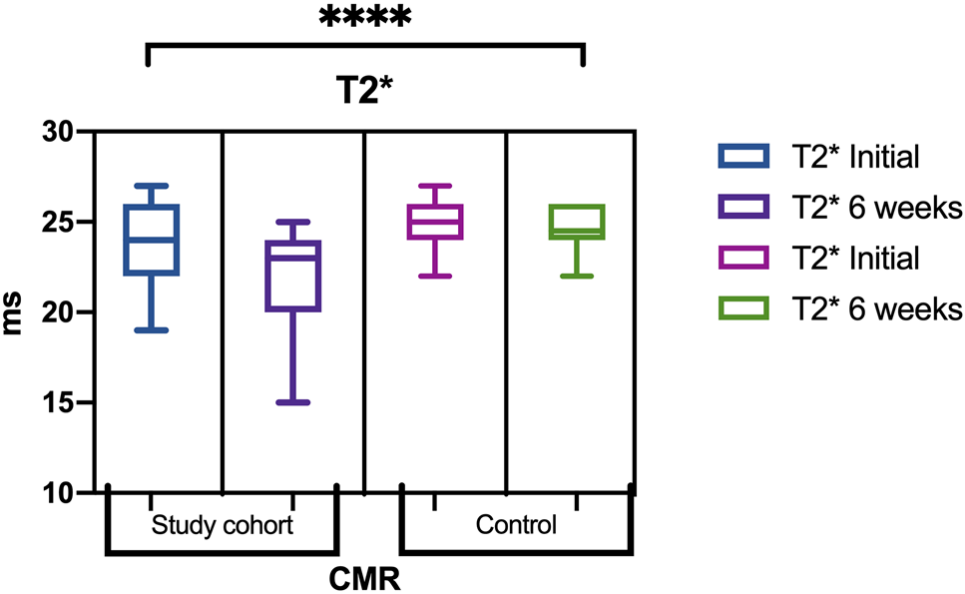
T2* Study cohort versus control subjects.

Regarding baseline myocardial iron levels and CMR assessment, in accordance with the normal range of myocardial T2* (95% confidence interval 20 ms), parameters of ventricular function (i.e., LVEF) fell within the normal confidence values (23 ± 1.97 ms)^30^ (Fig. 5). Corresponding to the 6-week CMR T2* determination according to the study protocol, there was a decrease in values (22.04 ± 2.80 ms) that was statistically significant; below a myocardial T2* value of 20 ms, we observed the onset of progressive and significant decline in the LVEF (r = 0.93, ****P < 0.0001).

**Figure 6:**
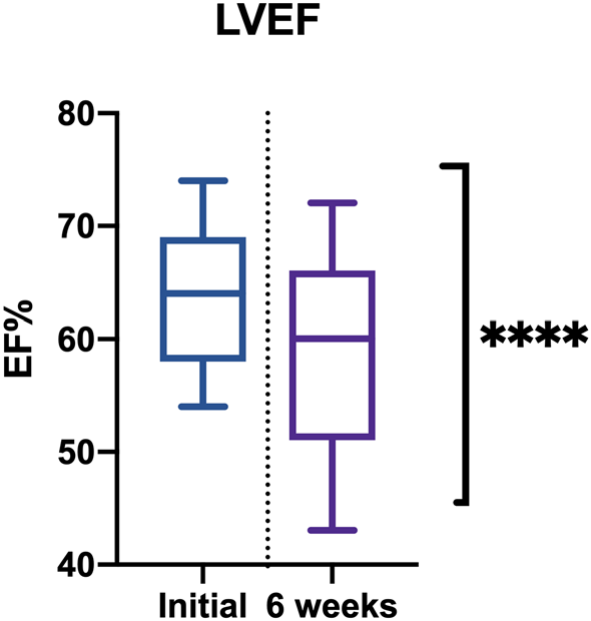
EF% at baseline and 6 weeks after treatment.

Dox-induced LV dysfunction with a decreased EF (< 50%) was found in 7 of 25 patients (28%) at the 6-week evaluation compared to that at the baseline evaluation, where only 2 patients presented a borderline EF% (55% and 54%) (Fig. 6).

Upon performing an in-depth evaluation of these cases, we determined that the incidence of myocardial abnormalities correspond elevated baseline values (well above average) for TLR4 expression and a large increase in transferrin levels (both sets of values having a logical correlation).

## Discussion

**Figure 7:**
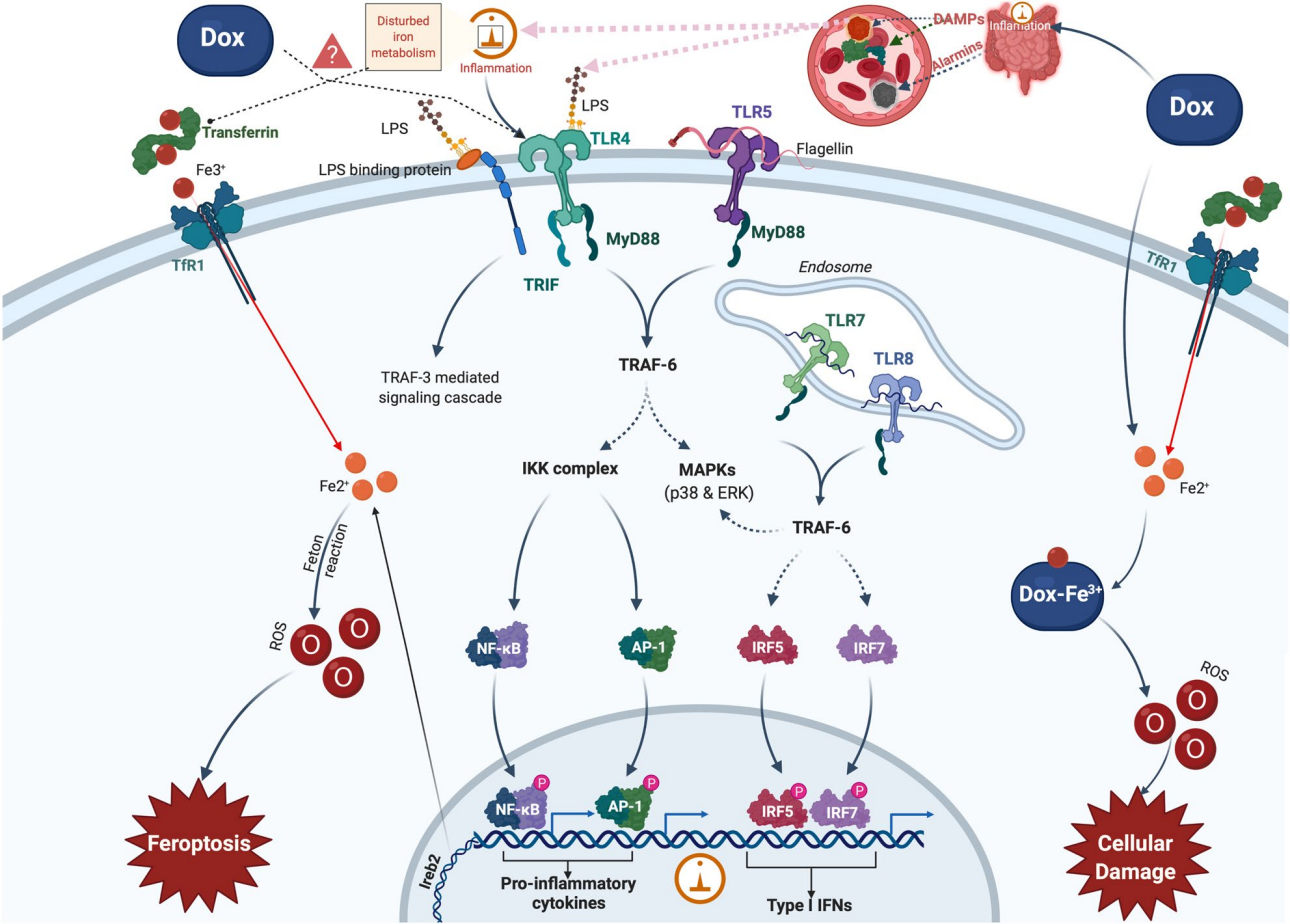
Schematic illustration of iron metabolism in human cells in the presence of doxorubicin. Diferric transferrin avidly binds to TfR1 on the cell membrane. The transferrin-TfR1 complex is then internalized into endosomes by receptor-mediated endocytosis. The iron is released from transferrin by the decrease in endosomal pH, which is mediated by a proton pump in the endosomal membrane. Once iron is released from transferrin, it is believed to be reduced by a ferrireductase and is then transported through the endosomal membrane into the cytoplasm by DMT1. Upon leaving the endosome, iron becomes part of a poorly characterized compartment known as the intracellular labile iron pool. Iron can be redistributed from the labile iron pool for cellular use, stored in ferritin, or potentially pumped out of the cell by ferroportin1. Doxorubicin and other anthracyclines bind iron to form the drug-iron(III) complex, which has been reported to produce ROS, which leads to cellular damage and apoptosis. TfR1—Transferrin receptor; ROS—reactive oxygen species; NF-kB—Nuclear Factor kappa-light-chain-enhancer of activated B cells.

In addition to forms of apoptotic cell death, which are already studied and well characterized, there are nonapoptotic forms of cell death that can trigger a systemic inflammatory through the release of DAMPs, which are to be recognized by IIRs^22^. The pathophysiological mechanisms that trigger cardiomyocyte dysfunction secondary to an iron overload process; however, are partially unknown (Fig. 7).

We postulate that the entry of an endotoxin into the circulation triggers TLR4-mediated systemic inflammation (an IIR early response). Endotoxin represents, in this case, a reasonable scenario comprising a key component for Gram-negative bacteria and a ligand of TLR4^31,32^. In normal homeostasis circumstances, a large number of bacteria presenting endotoxins reside in the intestine and are strictly confined by the barrier of the intestinal mucosa. However, this barrier could be broken by Dox, which is known to be capable of disrupting the epithelium to induce oral ulcers, intestinal inflammation, and hemorrhagic cystitis in cancer patients^33,34^. If Dox damages the intestinal mucosa, endotoxins could enter the circulation and stimulate systemic inflammation.

Furthermore, Dox was found to upregulate the expression of TLR2 and TLR4 in cardiomyocytes^35^. It is possible that this molecule will directly elevate the level of TLR4 in macrophages, resulting in stronger inflammatory responses to endotoxin and more severe damage to various organs.

Typically, significant LV dysfunction (more than 10% decrease for LVEF) has already occurred when CTX is detected by imaging techniques (with clinical manifestations). Biomarkers, most importantly cardiac natriuretic peptides and troponins, are promising markers for identifying patients potentially at risk for symptoms relating to clinical heart failure^36^. However, until now, these biomarkers have not proven to be practical as anthracycline-induced CTX screening and management tools.

Specifically, troponin was mainly demonstrated as a marker after administration of antineoplastic and β-sympathomimetic drugs, although routine use of these markers in monitoring patients receiving anthracycline therapy is far from being solved^37^.

Pre-existing comorbidities (e.g., high blood pressure, diabetes, hyperlipidemia) or unhealthy lifestyles (e.g., reduced physical activity) have long been known to increase the risk of CTX in patients scheduled to receive anthracyclines and were thus determined and analyzed within our batch study.

In our daily medical practice, identifying patients at increased risk of CTX is extremely important.

It should be noted that there is also a large individual variability of anthracycline sensitivity for each patient.

### Ferroptosis and TLR4 intercalation during Dox treatment

The term ferroptosis was first introduced in 2012 and was used to describe the form of cell death induced by a small molecule named erastin, which inhibits the import of cystine, leading to glutathione depletion and inactivation of the phospholipid peroxidase glutathione peroxidase 4 (GPX4)^38^.

**Figure 8:**
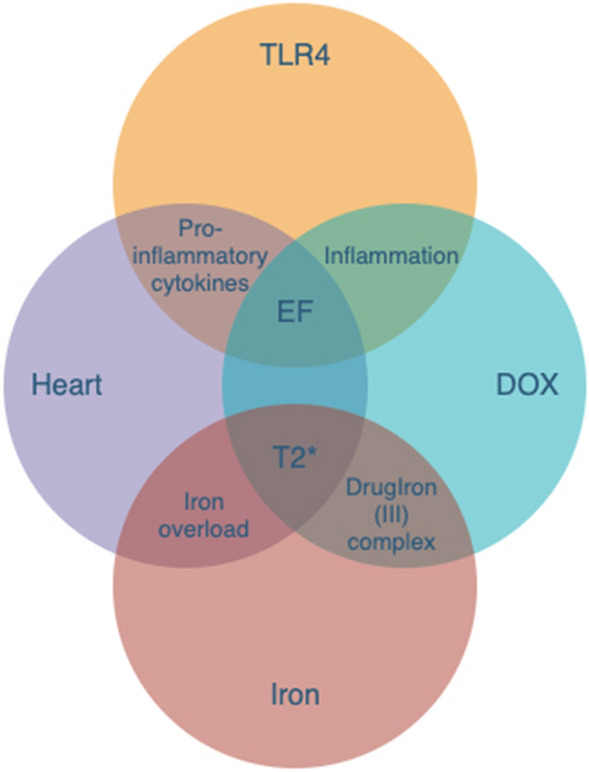
Venn diagram representation for interconnected and mutually amplifying inflammatory phenomena (Doxorubicin, TLR4, Iron and heart).

Our observations imply that endogenous substances released during ferroptotic cell death plays a key role in triggering TLR4 signaling in endothelial and cardiomyocyte cells. It is also more likely that ferroptosis results in the release of multiple DAMPs and other alarmin molecules that are recognized by a variety of cells within the heart, including immune cells, endothelial cells, and fibroblasts, thus triggering a cascade of interconnected and mutually amplified inflammatory phenomena (Fig. 8). It has become widely accepted that nonapoptotic forms of cardiomyocyte cell death result in the release of alarmins and DAMPs that ultimately initiate inflammation, and event known as necroinflammation.

Normally, iron overload occurs due to either increased supplementary gastrointestinal absorption or secondary to repetitive blood transfusions. Humans do not have a specifically adapted mechanism for excreting excess iron, which is usually deposited as crystalline iron oxide within ferritin and hemosiderin. The etiology of iron overload has consequences for iron tissue distribution. As an example, in hereditary hemochromatosis, iron is transported from the intestine to the liver via the portal vein (as transferrin) to be deposited in periportal hepatocytes. In other severe diseases with iron overload, iron is deposited in the pancreas and endocrine organs, especially in the heart^20,39^.

The data obtained from our study align with these facts, demonstrating a colinear interrelationship between TLR4 receptor expression and transferrin (r = 0.7628 and ****P < 0.0001) as a general reflection of an inflammatory process (both oncologically and in response to Dox as well as due to the activation of TLR4 receptors and the disruption of intra/extracellular iron homeostasis (especially in macrophages and the heart)).

Regarding the diagnosis of CTX using the EF, in the study group, 40% of patients who exhibited a decrease in the EF by more than 10% also had increased values (inversely proportional relationship) for increased genetic expressiveness of TLR4. Upon further exploring this relationship and causality, we deepened the correlation and analysis of the data and also found a decrease in T2* values (which reflects the iron load and cardiac sequestration and the onset of immediate cardiac dysfunction following treatment with Dox). By performing an in-depth evaluation of these cases, we determined that these changes corresponded to patients who initially expressed an increased TLR4 value (well above average) and a large increase in Transferrin (both sets of values having a logical correlation).

If we draw an even wider parallel at the level of TLR4 gene expression, and T2*, we see that there is a direct relationship reflected by an above average expressive level of the increased base of TLR4 and an above average decrease in times (ms) for T2*, which corresponds to immediate and rapid iron loading as a consequence of Dox treatment)*.*

## Conclusion

Our study showed a strong significant curvilinear correlation between genetic TLR4 expression and myocardial T2* (as obtained with the CMR technique) corresponding to iron overload in the heart as an immediate side effect of Dox treatment (most likely) through various mechanisms of action but with common effects.

EF, as an accepted marker of CTX, is also correlated in the whole table of the studied parameters, showing direct relationships with both TLR4 and T2*.

Risk stratification of Dox treatment with parameters already available to us can prove its usefulness in predicting the risk (immediate or acute) for the development of CTX as soon as possible; furthermore, more careful clinical follow-up and the initiation of possible treatments to counteract CTX can be implemented if screenings are conducted early in the disease course.
